# From prioritisation to understanding: mechanistic predictions of variant effects

**DOI:** 10.15252/msb.20188741

**Published:** 2018-12-20

**Authors:** Greg Slodkowicz, M Madan Babu

**Affiliations:** ^1^ MRC Laboratory of Molecular Biology Cambridge UK

**Keywords:** Chromatin, Epigenetics, Genomics & Functional Genomics, Computational Biology, Methods & Resources

## Abstract

The widespread application of sequencing technologies, used for example to obtain data from healthy individuals or patient cohorts, has led to the identification of numerous mutations, the effect of which remains largely unclear. Therefore, developing approaches allowing accurate *in‐silico* prediction of mutation effects is becoming increasingly important. In their recent study, Beltrao and colleagues (Wagih *et al*, 2018) describe an integrative approach for determining the effects of mutations from the perspective of protein structure, conservation and transcription factor binding. This allows for predicting the mechanisms underlying the most impactful variants rather than just identifying these variants.

New variants are now routinely being discovered by large‐scale sequencing of both healthy and patient cohorts, but understanding their phenotypic consequences remains a challenge. Existing methods can prioritise variants according to their potential to disrupt protein function, but these methods cannot uncover the mechanistic details underlying these disruptions. As a result, our understanding of the mechanisms underlying genetic diseases is lagging behind, despite the large number of existing prediction tools. In their recent work, Wagih *et al* (2018) present Mutfunc, a new resource that provides mechanistic predictions of mutational impact on function by combining sequence conservation with analysis of mutational effects on protein stability, interaction interfaces, post‐translational modifications and linear motifs. Additionally, Mutfunc predicts disruptions to start and stop codons, and quantifies the impact of non‐coding mutations, by analysing their effects on transcription factor binding sites, allowing predictions of gene expression deregulation. Mutfunc provides precomputed mechanistic annotations for mutations in three model organisms: *Homo sapiens*,* Saccharomyces cerevisiae* and *Escherichia coli* (Fig 1).

**Figure 1:**
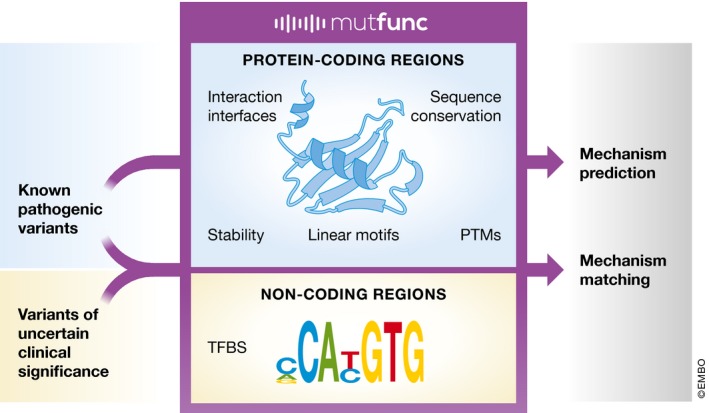
Mutfunc allows mechanistic predictions of variant effect for known pathogenic variants as well as variants of uncertain significance PTM, post‐translational modification; TFBS, transcription factor binding site.

Protein‐coding mutations can lead to a range of outcomes, from having no deleterious effect to causing diseases of moderate or high severity. In clinical studies, well‐described mutations are frequently outnumbered by uncharacterised variants discovered by high‐throughput sequencing of patient cohorts. Mechanistic predictions from Mutfunc can be used to identify variants with similar effects to those of already characterised variants and thus assign effects to variants with unknown clinical significance. For instance, known pathogenic variants in glyoxylate and hydroxypyruvate reductase (GRHPR) reduce its catalytic activity by disrupting homodimerisation. Applying Mutfunc to GRHPR variants with uncertain clinical significance reveals a subset of mutations that affect the homodimerisation interface in a manner similar to the confirmed disease mutations. Similarly, Mutfunc identifies variants in PARK2, a protein implicated in Parkinson's disease, which have a destabilising effect similar to that of known pathogenic variants. These variants are predicted to cause a disease phenotype. Wagih *et al* further validated Mutfunc by demonstrating a correlation between predicted functional impact and phenotype in 166 *S. cerevisiae* strains grown in 43 conditions. Combined per‐gene burden scores based on Mutfunc predictions were calculated, allowing the identification of a large number of significant associations between genotypes and growth rates.

These two types of validation highlight the usefulness of this approach in both focused studies aimed at separating neutral from deleterious variants, as well as in broader studies aimed at understanding the mechanistic details underlying previously identified genetic associations (e.g. in GWAS cohorts). Mutfunc can also be used to augment other genetic approaches, including polygenic risk scores which combine the predicted effects of many variants (Khera *et al*, 2018) without providing insights into the underlying disease mechanisms.

Historically, a major impediment to adopting methods based on protein structure was the significant shortage of solved structures. Mutfunc addresses this by providing high‐quality homology models, precomputed using the ModPipe software, greatly increasing structural coverage. Importantly, predicting mutational impact is particularly challenging within disordered regions. These regions lack a fixed conformation and generally do not appear in crystal structures. Currently, predictions are based on sequence conservation, disruptions in known linear motifs and post‐translational modification sites. Improving the ability to identify specific mechanisms by which mutations in disordered regions affect protein function, e.g. disorder‐to‐order transitions, would greatly increase the utility of mechanistic methods. In this context, experimental developments in large‐scale mutagenesis (Staller *et al*, 2018), phage display (Davey *et al*, 2017; Sundell *et al*, 2018), ligand foot‐printing and mass spectrometry (preprint: Parker *et al*, 2018) are beginning to provide insights into functional elements within disordered regions. Alongside these experimental approaches, hybrid experimental–computational approaches such as IDR‐Screen that exploit machine learning (Ravarani *et al*, 2018) will allow for new ways of uncovering mechanistic interpretation of mutations. Lastly, a more principled construction of multiple sequence alignments from which conservation is inferred can also improve predictions. In particular, distinguishing between orthologs and paralogs—which can be subject to very different evolutionary constraints—has been shown to improve variant effect prediction in disease context (Adebali *et al*, 2016), as well as the identification of specificity‐determining residues whose functional importance may be different among paralogs (Flock *et al*, 2017).

On the basis of the analysed structural features, the set of variant effect predictions from Mutfunc allowed Wagih *et al* to demonstrate that important functional regions of proteins are depleted in naturally occurring variants. Understanding the constraints imposed by protein structure is a fundamental question in evolutionary biology, and these insights are important both for theoretical and practical reasons. Aggregation of single nucleotide polymorphism (SNP) data can be used to identify constrained regions in proteins (preprint: Samocha *et al* 2017), and recent work on the structural placement of variants demonstrated that, while naturally occurring SNPs tend to be dispersed on protein structures, pathogenic mutations are more likely to be clustered (Sivley *et al*, 2018). Approaches based on clustering in sequence or in space can thus be used to identify new structural and functional features that can then be integrated into the methods predicting the mechanistic impact of mutations.

Most existing variant effect prediction methods fail to distinguish between loss‐ and gain‐of‐function mutations. Mechanistically distinguishing between these two effects would be a significant advancement and would also help identify dominant‐negative or haploinsufficiency effects that can cause variants to have different penetrance. The effect of deleterious mutations can also be masked by compensatory effects (epistasis) and allele‐specific expression or it can manifest upon environmental influences, including lifestyle choices. Currently, neither mechanistic nor other kinds of prediction tools can directly account for incomplete penetrance of mutations. Overcoming these limitations will require integrating predictions and findings across different levels of complexity, from mutational signatures and their mechanistic effects, to molecular phenotypes, cellular interaction networks and environmental influences.

We are entering a particularly exciting time as, in addition to sequence variation, other data types such as single‐cell gene expression, structural, interaction, epigenetics, metabolomics, transcriptomics and proteomics data are becoming increasingly available. Integration of high‐quality unbiased data spanning different levels of biological organisation will be key for advancing our understanding of genotype‐to‐phenotype relationships, including disease mechanisms.

## References

[msb188741-bib-0001] Adebali O , Reznik AO , Ory DS , Zhulin IB (2016) Establishing the precise evolutionary history of a gene improves prediction of disease‐causing missense mutations. Genet Med 18: 1029–1036 26890452 10.1038/gim.2015.208 PMC4990510

[msb188741-bib-0002] Davey NE , Seo MH , Yadav VK , Jeon J , Nim S , Krystkowiak I , Blikstad C , Dong D , Markova N , Kim PM , Ivarsson Y (2017) Discovery of short linear motif‐mediated interactions through phage display of intrinsically disordered regions of the human proteome. FEBS J 284: 485–498 28002650 10.1111/febs.13995

[msb188741-bib-0003] Flock T , Hauser AS , Lund N , Gloriam DE , Balaji S , Babu MM (2017) Selectivity determinants of GPCR–G‐protein binding. Nature 545: 317 28489817 10.1038/nature22070 PMC5846738

[msb188741-bib-0004] Khera AV , Chaffin M , Aragam KG , Haas ME , Roselli C , Choi SH , Natarajan P , Lander ES , Lubitz SA , Ellinor PT , Kathiresan S (2018) Genome‐wide polygenic scores for common diseases identify individuals with risk equivalent to monogenic mutations. Nat Genet 50: 1219 30104762 10.1038/s41588-018-0183-z PMC6128408

[msb188741-bib-0005] Parker B , Goncz E , Krist DT , Statsyuk A , Nesvizhskii AI , Weiss E (2018) LiF‐MS: mapping unstructured peptide‐protein interactions using Ligand‐Footprinting Mass Spectrometry. bioRxiv 10.1101/361857 [PREPRINT] PMC6800362 31578253

[msb188741-bib-0006] Ravarani CN , Erkina TY , De Baets G , Dudman DC , Erkine AM , Babu MM (2018) High‐throughput discovery of functional disordered regions: investigation of transactivation domains. Mol Syst Biol 14: e8190 29759983 10.15252/msb.20188190 PMC5949888

[msb188741-bib-0100] Samocha KE , Kosmicki JA , Karczewski KJ , O'Donnell‐Luria AH , Pierce‐Hoffman E , MacArthur DG , Neale BM , Daly MJ (2017) Regional missense constraint improves variant deleteriousness prediction. bioRxiv 10.1101/148353 [PREPRINT]

[msb188741-bib-0007] Sivley RM , Dou X , Meiler J , Bush WS , Capra JA (2018) Comprehensive analysis of constraint on the spatial distribution of missense variants in human protein structures. Am J Hum Genet 102: 415–426 29455857 10.1016/j.ajhg.2018.01.017 PMC5985282

[msb188741-bib-0008] Staller MV , Holehouse AS , Swain‐Lenz D , Das RK , Pappu RV , Cohen BA (2018) A high‐throughput mutational scan of an intrinsically disordered acidic transcriptional activation domain. Cell Syst 6: 444–455 29525204 10.1016/j.cels.2018.01.015 PMC5920710

[msb188741-bib-0009] Sundell GN , Arnold R , Ali M , Naksukpaiboon P , Orts J , Güntert P , Chi CN , Ivarsson Y (2018) Proteome‐wide analysis of phospho‐regulated PDZ domain interactions. Mol Syst Biol 14: e8129 30126976 10.15252/msb.20178129 PMC6100724

[msb188741-bib-0010] Wagih O , Galardini M , Busby B , Memon D , Typas A , Beltrao P (2018) A resource of variant effect predictions of single nucleotide variants in model organisms. Mol Syst Biol 14: e8430 10.15252/msb.20188430 PMC6301329 30573687

